# Advances in 4-Nitrophenol Detection and Reduction Methods and Mechanisms: An Updated Review

**DOI:** 10.1021/acsomega.4c04185

**Published:** 2024-07-25

**Authors:** Alan Omar Cardoso Juarez, Edgar Ivan Ocampo Lopez, Mohan Kumar Kesarla, Naveen Kumar Reddy Bogireddy

**Affiliations:** Instituto de Ciencias Físicas (ICF), Universidad Nacional Autónoma de Mexico (UNAM), Avenida Universidad 1001, Col. Chamilpa, Cuernavaca C.P 62210, Morelos, Mexico

## Abstract

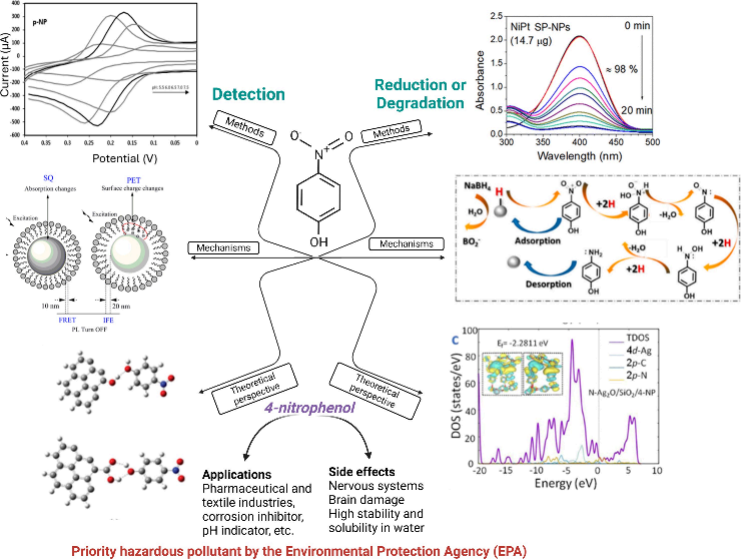

This review emphasizes the progress in identifying and eliminating para-nitrophenol (4-NP), a toxic organic compound. It covers various strategical methods and materials, including organic and inorganic nanomaterials, for detecting and reducing 4-NP. Detection techniques such as electrochemical methods. Optical fiber-based surface plasmon resonance and photoluminescence, as well as the mechanisms of Förster Resonance Energy Transfer (FRET) and Inner Filter Effect (IFE) in fluorescence detection, are presented. Removal techniques for this contaminant include homogeneous catalysis, electrocatalysis, photocatalysis, and thermocatalysis, and their reaction mechanisms are also discussed. Further, the theoretical perspectives of 4-NP detection and reduction, parameters influencing the activities, and future perspectives are also reviewed in detail.

## 1 Introduction

In recent decades, humanity has faced a series of global problems that affect the economy, health, lifestyle, and life of living beings. Among the most worrying problems are poverty and hunger in many parts of the world (countries, regions, and states). In the same way, pollution and access to drinking water are among the five most worrying problems. To deal with these last two problems mentioned above, different teams and research groups from several countries focused on developing, modifying, creating, and innovating synthesis methods, materials at different size scales, characterization techniques, as well as detection, reduction, and degradation processes of contaminants present in soils, air, and aqueous bodies. Heavy metals, dyes, industrial waste, pesticides, fertilizers, and phenols are the contaminants that generate the most significant interest and concern. Several persistent organic pollutants are known to cause harm to the health of living beings and the environment, even at low concentrations. These contaminants can resist natural degradation caused by the sun, chemical agents, and microorganisms over many years.^1,2^

4-Nitrophenol (4-NP) or p-nitrophenol (C_6_H_5_NO_3_) is a phenolic compound and a pale-yellow crystalline material. It is used as a colorless pH indicator at pH less than 5.4 and yellow at pH greater than 7.5 due to a deprotonated state (4-nitrophenolate ion). It is also used for the synthesis of different pharmaceutical substances of great importance to health, such as paracetamol (analgesic and antipyretic), phenetidine (anticonvulsant), and acetophenetidine (analgesic), as well as for the manufacture of insecticides, fungicides, and dyes to darken the leather.^3,4^ The World Health Organization (WHO) and the Environmental Protection Agency (EPA, U.S.) designated 4-NP as a highly hazardous chemical, establishing a permissible limit in drinking water of 1 μg/L according to Mexican NOM-127-SSA1–1994^5^ due to its high or prolonged exposure to 4-NP could affect the nervous system and interfere with the blood’s ability to carry oxygen.^6^ Additionally, inhalation of 4-NP not only causes breathing difficulties, such as irritation of the nose, throat, and lungs, cough, or shortness of breath, but may also cause upset stomach, weakness, rapid heartbeat, confusion, or fever,^7^ even causing collapse and death.^8^

The identification of 4-NP is commonly performed by electrochemical methods or high-performance liquid chromatography,^9,10^ which are relatively expensive, complex, and poorly accessible techniques. Interest has been aroused in developing new analytical tools. Portable devices can detect 4-NP in a highly selective, sensitive, and rapid manner. In recent years, various nanomaterials have been evaluated, such as CdTe,^11^ CdS,^12^ CdSe,^13^ gold nanoparticles (AuNPs),^14^ copper nanoparticles (CuNPs),^15^ and graphene oxide/carbon dots (CDs).^1,16^ Detecting 4-NP using fluorescent nanomaterials such as carbon dots is exciting due to its environmentally friendly nature and economic efficiency, which involves the extinction of the emitted light (when exposed to higher energy electromagnetic radiation^17^ in the presence of the contaminant. The reduction or degradation of various pollutants present in the air, soil, or water bodies is usually carried out through catalysis or electrochemical processes due to their high efficiency, effectiveness, reduced costs, and achievable reaction conditions.

A few reports on reducing 4-NP,^18,19^ including our recent report,^20^ focused mainly on the biogenic synthesis of nanostructured catalysts to reduce nitrophenol. We know that no specific review article on combined detection and reduction exists. This review addresses the problems associated with 4-NP, a relevant contaminant that impacts human health and the environment. The importance of this compound, its adverse effects, and the identification and removal methods used in its detection and reduction are analyzed. Additionally, the advances in detection techniques and reaction mechanisms used in the reduction/degradation of 4-nitrophenol are discussed. A theoretical perspective is presented, and the parameters that influence the detection and reduction of this contaminant are discussed to offer a comprehensive and updated vision of this critical environmental research topic.

## 2 4-Nitrophenol Detection

### 2.1 Organic Materials as 4-NP Detectors

Particularly, through various organic precursors, various groups have been investigating the ability of carbon dots (CDs) to be selective against 4-NP. For example, CDs obtained from natural celery leaves and glutathione (N, S dopants) (Qu et al.^21^) demonstrated their selectivity toward 4-NP, 2-nitrophenol (2-NP), and 3- 3-nitrophenol (3-NP). In another study, CDs synthesized from citric acid and urea (Bogireddy et al.^1^) showed selectivity toward 4-NP. Das and Dutta synthesized CDs selectively to 4-NP using ethylene glycol and β-alanine.^22^ On the other hand, Hu and Gao^23^ made CDs using wastewater sludge as precursors, and they were selective for 4-NP. Tu et al.^24^ synthesized CDs using Auricularia auricula as precursors, being selective for 4-NP. In the study by Tu et al.,^25^ CDs were generated using *Ganoderma lucidum* (Gl) spore powder-mediated pristine CDs, N-doped, and phosphorus-doped CDs showed selectivity toward 4-NP and DNP. Further, Liao et al.^26^ and Omama et al.^27^ used hexamethylenetetramine and triphenylamine to make CDs and tested for the 4-NP detection. Other materials used to sense 4-NP include fluorescent poly(ionic liquid) based on coumarin^28^ and fluorescent molecular imprinted sensor based on fluorescent polydopamine,^29^ coffee,^30^ and rice.^31^

The detection and sensing of 4-NP is a topic of interest for several researchers who have been exploring different approaches using organic and inorganic materials. In the case of organic materials, it has been observed that carbon dots (CDs) derived from various organic precursors show selectivity toward 4-NP. For example, studies have shown that CDs obtained from natural celery leaves and glutathione, citric acid and urea, ethylene glycol, and β-alanine, among others, exhibit selectivity toward 4-NP. These CDs have been synthesized using hydrothermal, oligomerization, and microwaves, and luminescence is used as the primary sensing mechanism. Detection limits vary depending on the material and synthesis method, ranging from 0.2 μM to 0.5 nM (Table 1).

**Table 1:** Organic nanomaterials for the detection of 4-NP with photoluminescence technique

**Sensing probe**	**Synthesis method**	**Detection limit (nM)**	**Ref**
Citric acid and urea	Hydrothermal	2000	Bogireddy et al.^1^
Celery leaves and glutathione	Hydrothermal	26 and 100	Qu et al.^21^
Ethylene glycol and β-alanine	Oligomerization	400	Das and Dutta^22^
sewage sludge	Microwave	69	Hu & Gao^23^
Auricularia auricle	Hydrothermal	198	Tu et al.^24^
Ganoderma Lucidum Spore Powder	Hydrothermal	68	Tu et al.^25^
Hexamethylenetetramine	Hydrothermal	201	Liao et al.^26^
Triphenylamine	Agitation with temperature	79	Omama et al.^27^
Coumarin	Molecular print	0.5	Dai et al.^28^
Fluorescent poly dopamine	Molecular print	24.2	Xu et al.^29^
Coffee	Carbonization	10.9	Baye at al.^30^
Marine Rice	Carbonization	34	Luo et al.^31^

### 2.2 Inorganic Materials as 4-NP Detectors

The use of inorganic materials for detecting 4-NP is usually by electrochemical methods, although it is not limited to that method. Among the materials used are silicon nanoparticles,^32^ organometallic structures,^33^ polyaniline/platinum optical fibers,^34^ using lanthanides,^35^ reduced graphene oxide (rGO), and halloysite nanotubes (HNT) with silver nanoparticles (AgNPs),^36^ glassy carbon modified with the graphene-chitosan composite film,^37^ copper-doped CeO_2_ nanoparticles^38^ and Cu–Fe_3_O_4_ nanocomposite.^39^ Zeolitic imidazolate-8 combined with carbon dots,^40^ trisodium citrate with 3-aAminopropyltriethoxysilane,^41^ Mn–Fe_3_O_4_ anchored to graphene,^42^ Ag_2_O-ZnO nanocones,^43^ Au/CaCO_3_,^44^ Ni/Cu_2_O,^45^ SrSnO_3_ and conductive polymers,^46^ and Co_3_O_4_.^47^

Inorganic materials, such as silicon nanoparticles, organometallic structures, and polyaniline/platinum optical fibers, are also effective to detect 4-nitrophenol. These materials usually test electrochemical methods for sensing, although other methods have also been used, such as surface plasmon resonance. The detection limits for these inorganic materials vary from 0.076 μM to 0.034 pM, dependent to the material and the fabrication procedure used (Table 2).

**Table 2:** Inorganic Nanomaterials for the Detection of 4-Nitrophenol

**Sensing probe**	**Synthesis Technique**	**Detection method**	**Detection limit (nM)**	**Ref**
Si	Agitation with temperature	Luminescence	74	Liu et al.^32^
Amine-UiO-66/MIP	Molecular print	Luminescence	0.009	Amiripour et al.^33^
Polyaniline/platinum	Cathodic pulverization	Surface plasmon resonance	0.00034	Antohe et al.^34^
LZG-Eu y LZG-Tb	Solvothermal	Luminescence	11.2	Lin et al.^35^
rGO-HNT-AgNP	Oxidation	Electrochemical	48.6	Hwa et al.^36^
Chitosan-graphene	Agitation	Electrochemical	57	Yin et al.^37^
CeO_2_ y Cu	Coprecipitation	Electrochemical	NR^a^	Ansari et al.^38^
Cu–Fe_3_O_4_	Hydrothermal	Electrochemical	65	Cao et al.^39^
CDs@ZIF-8	Molecular imprint	Luminescence	50	Fua et al.^40^
APTES y sodium citrate	Hydrothermal	Luminescence	23.45	Zhu et al.^41^
Mn–Fe_3_O_4_ and graphene	Solvothermal	Electrochemical	19	Su et al.^42^
Ag_2_O-ZnO	Microwave	Electrochemical	23	Chakraborty et al.^43^
Au/CaCO_3_	Agitation with temperature	Electrochemical	0.54	Ding et al.^44^
Ni/Cu_2_O	Chemical deposition	Electrochemical	16.0	Pang et al.^45^
SrSnO_3_	Carbonization and oxidative polymerization	Electrochemical	0.15	Katowah et al.^46^
*Bauhinia vahlii* and Co_3_O_4_	Hydrothermal	Electrochemical	5	Benadict et al.^47^

a NR = Not reported.

In summary, organic and inorganic materials have shown promise to detect 4-NP selectively, each with advantages and detection limits. Developing and optimizing these materials and detection methods are constantly evolving research areas in analytical chemistry and sensitization.

## 3 Detection Methods and Mechanisms

### 3.1 Electrochemical Technique

**Figure 1:**
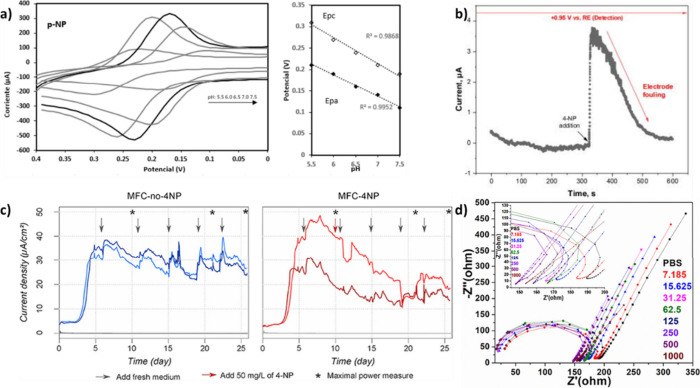
Electrochemical techniques for 4-nitrophenol detection: (a) CV curves show the current and potential change as a function of pH (0.1 M PBS),^49^ (b) 4-NP detection in the constant potential mode of amperometry,^50^ (c) current density produced by catalyst modified with 4-nitrophenol,^51^ and (d) 4-NP concentration dependent EIS responses in buffer solution.^38^

Electrochemical tests are analytical methods that use the electrical response of a system to identify and measure the existence of contaminants in a water sample.^48^ The mechanism can be exemplified in Figure 1. Some common types of electrochemical tests used to detect contaminants in water are as follows.

#### Voltammetry

In voltammetry, an electrical signal is measured as a function of time.^36,49^ The resulting waveform, a voltammogram, can provide information about the analytes present in the sample. Voltammetry includes techniques such as differential pulse voltammetry and CV, as shown in Figure 1 (a). In his doctoral thesis,^49^ Ayala determined 4-NP concentration as a function of pH using modified electrodes with graphite and gold and was also able to obtain a minimum identification limit of 1.5 μM. Ansari et al.^38^ improved copper-doped cerium oxide nanoparticles prepared by a polyol-assisted coprecipitation process, where, using electrochemical techniques, they could detect 4-NP with a sensitivity of 1.4 μA/mM.

#### Amperometry

In amperometry, the electric current produced by the redox consequence of the analytes extant in the sample is measured.^47^ This method is beneficial for detecting substances that may be electroactive, such as some organic contaminants.

#### Potentiometry

The electrical potential is determined without applying current, it is to measure the specific ion concentrations in the experiment, such as the pH of water (Figure 1(b)), where Prempininj et al.^50^ used electrodes from a 3 mm diameter boron-doped diamond disk, 3 M Ag/AgCl/KCl, and a Pt wire electrode, and a scanning speed of 100 mV s^–1^, obtaining a sensitivity to 10 μM.

#### Electrochemical Biosensors

These devices use biological systems (such as enzymes, antibodies, cells, or biomolecules) together with electrochemical techniques to specifically detect specific contaminants.^37^ The interaction between the analyte and the biological component produces detectable changes in current or potential. Yin et al.^37^ synthesized GCE coated with a graphene-chitosan complex to make an electrode sensitive to 4-NP and obtained a detection limit of 0.057 μM.

#### Fuel Cells

Although primarily used for power generation, fuel cells have also been applied to detect contaminants in water.^51^ Specific analytes can affect cell efficiency and can be used as an indicator of contamination, as shown in Figure 1(c), where Godain et al.^51^ used microbial fuel cells (MFC) they worked with synthetic effluent to mimic practical environment as allowing control of TOC levels. The voltage of each catalyst was noted, and they recorded the degradation rate of 4-NP with 36 mg/h.

#### Electrochemical Impedance Sensors

These sensors measure the electrical impedance of a system in response to the application of a small excitation voltage.^38,39^ Changes in impedance due to interaction with analytes can provide information on the concentration and nature of contaminants, as shown in Figure 1(d). Ansari et al.^38^ developed copper-doped cerium oxide nanoparticles prepared by a polyol-assisted coprecipitation process, where, using electrochemical techniques, they could detect 4-NP with a sensitivity of 1.4 μA/mM.

The methods and mechanisms for detecting contaminants in water (highlighting 4-NP for this work) cover a wide range of analytical techniques, each with specific advantages and applications. Among the electrochemical methods mentioned, voltammetry, amperometry, potentiometry, electrochemical biosensors, and electrochemical impedance sensors stand out. These methods take advantage of the electrical response of systems to detect and quantify the presence of contaminants, offering sensitivity and selectivity in detecting various analytes, including pesticides, heavy metals, specific ions, and organic compounds. Electrochemical tests are often combined with traditional techniques to obtain precise and accurate results.

### 3.2 Photoluminescence-Based Detection

**Figure 2:**
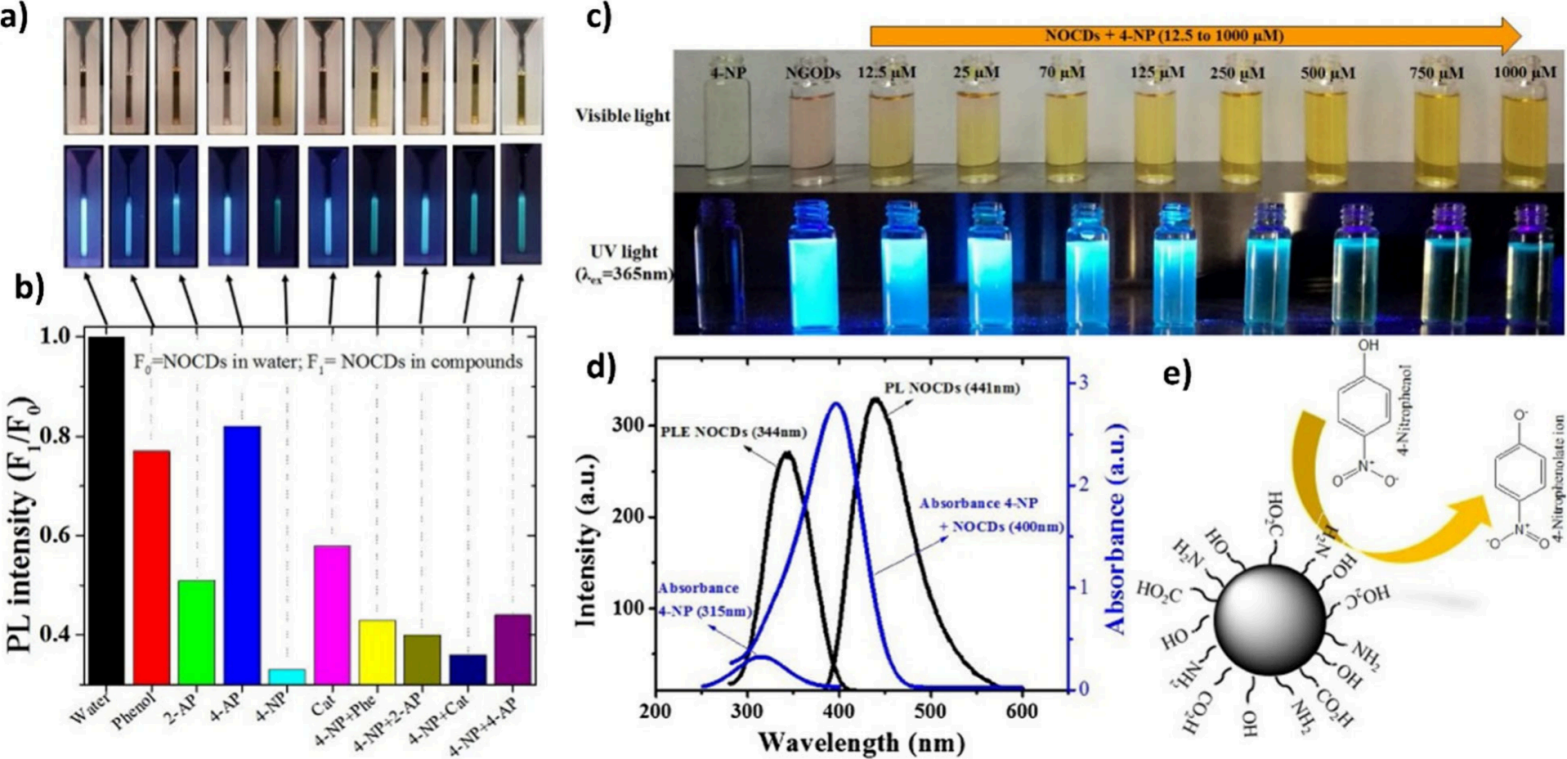
Fluorescent carbon dots in different contaminants (a) photogenic representations under daylight/UV lamp (365 nm), (b) normalized PL signal of CDs in contaminants. (c) photographic images of CDs with the function of 4-NP concentrations from 0.0125 μM to 1 mM solution, (d) absorbance, PL, and PLE analysis of CDs without and with the presence of 4-NP, and (e) pictorial illustration of 4-NP sensing mechanism. Adopted with permission from ref (1). Copyright 2020 Elsevier.

Photoluminescence selectivity refers to tests or analytical techniques that use the emission of light (photoluminescence) from a sample to identify and quantify certain compounds. This approach is advantageous in detecting specific substances that exhibit light-emitting properties when excited by an energy source, such as ultraviolet (UV) light. Photoluminescence involves the absorption of energy (e.g., by ultraviolet light) by specific compounds in the sample, followed by light emission when these compounds return to their lower energy state.^1,57,58^ Light emission can be detected and measured, providing information on the presence and concentration of compounds of interest, as shown in Figure 2. Our group^1^ synthesized carbon dots selective to 4-NP using citric acid and urea, obtaining a detection limit of 2 μM. Other authors who have carried out 4-NP detection by luminescence reported detection limits in the range of nanomolar and micromolar (Figure 2). For example, Qu et al.^21^ obtained 26 nM and 0.1 μM by synthesizing carbon dots using celery leaves and glutathione; Hu and Gao^23^ reported a detection limit of 69 nM using sewage sludge as a precursor.

In many applications, specific fluorescent labels selectively bind or react with the analytes of interest. These markers emit light when excited. Selectivity is achieved using markers with an affinity for certain compounds or chemical groups. Photoluminescence selectivity tests are applied in various areas, including detecting contaminants in water, food, and environmental samples. Specific contaminants can be detected depending on the selectivity of the marker used. Among the advantages is its high sensitivity; photoluminescence often allows the detection of low levels of analytes. The choice of specific fluorescent markers allows the selective identification of compounds of interest; some photoluminescence tests can provide rapid and real-time results. On the other hand, interferences from other compounds present in the sample may arise as part of this technique’s challenges. Furthermore, the stability and selectivity of the markers used are critical factors.

Photoluminescence selectivity tests are valuable tools in detecting and quantifying specific contaminants. They are used in various disciplines, from scientific research to environmental monitoring and food safety.

Förster resonance energy transfer (FRET), also known as nonradiative energy, is a quantum phenomenon that describes nonradiative energy transfer of energy among two chromophores when they are in proximity, and their absorption and emission spectra overlap.^50,59,60^ When a “donor” chromophore in an excited state transfers energy to another nearby “acceptor” chromophore in a ground state. The 4-nitrophenol can quench the signal of PL of the CDs through the FRET mechanism, because the 4-nitrophenol absorption band overlaps with the PL excitation band of the CDs and, the PL lifetime change of the CDs in the presence of p-NP is also noted.^26^ The proximity of the chromophores and the change in FRET efficiency may indicate changes in the surrounding environment or the molecule’s conformation.

### 3.3 Surface Plasmon Resonance-Based Detection

**Figure 3:**
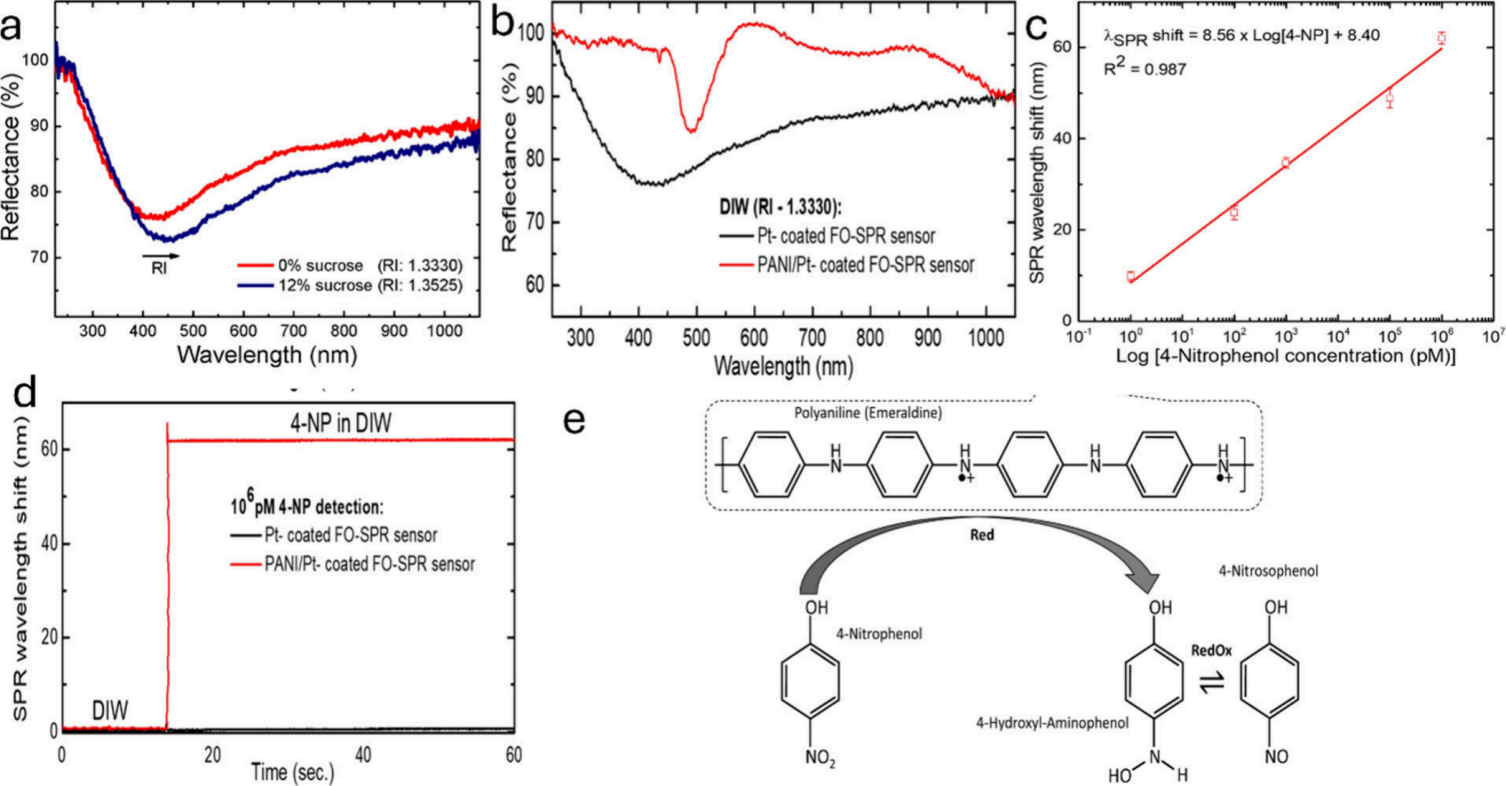
Fiber optic SPR-based sensors: (a, b) SPR spectra obtained at 0 and 12% sucrose concentrations and with Pt-coated FO-SPR and PANI/Pt detectors, respectively. (c, d) Analysis graph of 4-NP concentrations revealing a linear tendency among the wavelength shift vs logarithm with the absence of the PANI layer, and (e) proposed 4-NP sensing and reduction mechanisms. Adapted with permission from ref (34). Copyright 2021 Nature Publishing Group.

Surface plasmon resonance with optical fiber is a phenomenon that occurs when light couples with a surface plasmon in an optical fiber.^34,63−65^ Surface plasmons are electronic waves propagating along the interface between a conductive material, such as metal, and a dielectric, such as air or a liquid. Light can be coupled with surface plasmons. When it hits this interface at a specific angle and wavelength, it results in significant light absorption. In the context of optical fiber, this implies that light propagating along the fiber can interact with surface plasmons on the outer surface of the fiber. This phenomenon can be exploited to perform measurements sensitive to changes in the environment close to the optical fiber, such as detecting biomolecules, gases, or changes in the concentration of chemical substances, as shown in Figure 3. As shown by the team of Antohe et al.,^34^ the polyaniline/platinum optical fibers synthesized by sputtering could detect 4-NP minimum detection limit of 0.34 pM. Highlighting that platinum and polyaniline (PANI) were chosen for this study due to their unique and complementary properties. Platinum was selected as the plasmonic material to coat the optical fiber because of its excellent catalytic properties, stability, and conductivity. On the other hand, PANI was chosen as the sensing material for detecting 4-nitrophenol due to its stability, favorable physicochemical properties, and ability to react with various molecular species. Combining these two materials allowed for the fabrication of a highly sensitive and efficient sensor for detecting traces of 4-nitrophenol in water samples.

The process of detecting 4-NP involves PANI playing a central role, where sites terminated with H^+^ ions initiate the conversion of 4-NP into 4-hydroxyl-aminophenol, which is then oxidized to form 4-nitrosophenol leads to a Redox reaction facilitated by the catalytic properties of the PANI/Pt bilayer, resulting in significant alterations in the refractive index of the surrounding medium due to the conversion of 4-NP into 4-nitrosophenol. Consequently, there are noticeable shifts in the wavelength position of SPR spectral dips. These impressive performance attributes are also credited to the optimal thickness of PANI and its textured, spiral-like surface morphology, which amplifies the active surface area of the FO-SPR sensor, potentially improving the efficiency of catalytic surface reactions between 4-NP and the PANI layer.

Surface plasmon resonance with optical fiber stands out as a phenomenon that allows measurements that are sensitive to changes in the environment close to the optical fiber, resulting in the detection of biomolecules, gases, or changes in the concentration of chemical substances.

Together, these techniques offer a variety of approaches for the detection and quantification of 4-NP in water, allowing researchers and environmental professionals to obtain precise and accurate results for evaluating and monitoring water quality. The choice of the appropriate technique will depend on the nature of the contaminants and the specific objectives of the analysis, and several techniques can be combined to obtain a more exhaustive evaluation.

After comparing the different methods and materials, we observe distinct characteristics for each. Electrochemical techniques offer high selectivity in detecting contaminants like 4-NP through specific electrode modifications and reactions. Electrochemical methods are generally cost-effective due to the availability of standard electrodes and simple setups, and they have demonstrated high sensitivity with detection limits ranging from 0.15 nM to 5 nM for 4-NP. However, the downside is that specific electrodes or modifications may be required for different analytes, which can increase complexity.

On the other hand, photoluminescence-based methods offer different advantages. They are highly selective in detecting compounds through their absorbance and PL properties and adaptable by utilizing organic waste as a primary carbon source. This makes them the simple procedure, cost-effective, and eco-friendly. They also exhibited the broad detection limits range from nM to μM for 4-NP.

Meanwhile, the Surface Plasmon Resonance (SPR) methods, with their high selectivity by detecting changes in the environment close to the optical fiber, offer a different level of sensitivity. SPR has shown minimum detection limits of 0.34 pM for 4-NP, a testament to its high sensitivity. However, SPR from optical fiber may require specialized equipment. It can provide highly selective and sensitive detection, potentially justifying the cost.

Therefore, if high selectivity is the primary concern, all three methods offer selective detection capabilities for 4-NP. Photoluminescence and SPR methods may suit specific applications requiring high sensitivity and selectivity. However, the photoluminescence method is the most suitable, considering its cost-effectiveness, nontoxicity, possible on-site measurement use, and high efficiency. It can be made portable, and the materials used for this method are economical.

### 3.4 Theoretical Perspective

**Figure 4:**
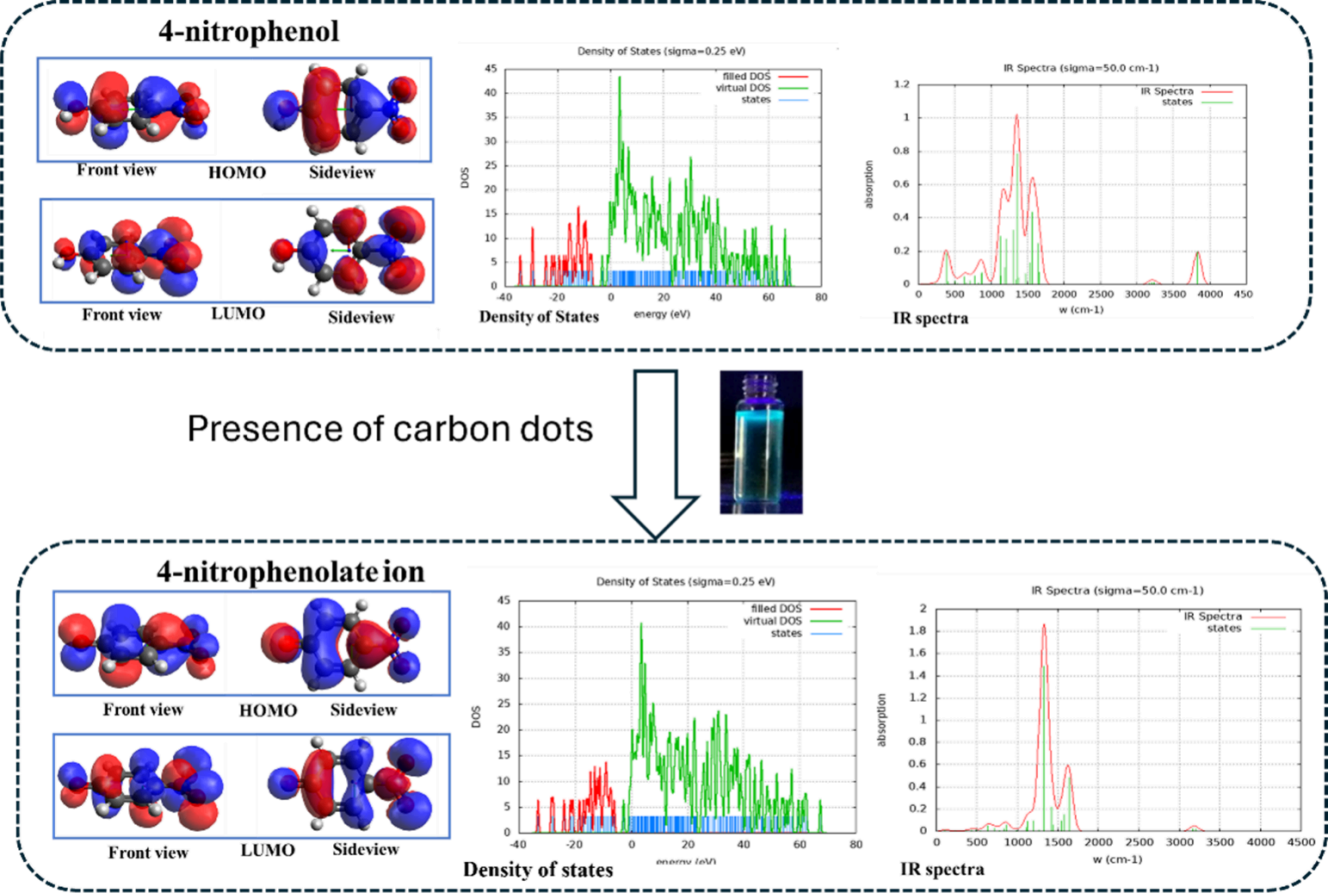
Quantum chemical computations such as Frontier molecular view, HOMO–LUMO, density of states, and IR spectra related to 4-NP (upper part) and CDs reacted to 4-NP (i.e., 4-nitrophenolate ion (lower part)). Adopted with permission from ref (1). Copyright 2020 Elsevier.

Although carbon dots have been produced using three main dopants (N/S/P),^1^ a preferred dopant for 4-NP detection has yet to be comprehensively investigated and established (Figure 4). Instead, the choice of dopant type and concentration has been quite random. Furthermore, a few theoretical/experimental experiments have been performed to recognize the interaction process of 4-NP with carbon dots. For example, Wang Z et al.^66^ analyzed the effect of different initial concentrations of 4-NP, initial concentrations of reductant, catalyst dosage, and reaction temperatures to understand the adsorption followed by degradation of 4-nitrophenol through the Dmol3 code. Aola Supong et al.,^67^ employing the B3LYP level, reported that carboxyl groups in bioactivated carbon interact significantly with 4-NP, and recently, our group^68^ detailed the role of pyridine N in the reduction of 4-NP. Given the importance of heteroatom studies on the interaction of 4-NP with the dopant and its relative concentration in the N-CDs, it is time to use advanced computing techniques through theoretical chemistry methods, which enable the simulation of molecular models and electronic structures to calculate the properties of these systems. The effectiveness of these computational tools has been evidenced by correlating the results obtained from the models with experimental results.^69^

## 4 4-Nitrophenol Reduction

Researchers have been developing and studying the 4-NP reduction in recent years. Several types of materials can be classified in different ways, such as sustainable and nonsustainable. Although the concept of sustainability has been evolving, it is attributed to the fact that sustainable nanomaterials have a beneficial social, economic, and environmental impact. The classification of nanomaterials can be defined depending on the focus of interest; in this case, the classification that depends on the origin of the raw materials will be used, as well as the synthesis processes of each of these, which would be organic and inorganic.

There are many methods and processes for the synthesis of nanomaterials, each with its virtues and disadvantages. This is one of the main aspects to consider determining whether a nanomaterial is sustainable or not. Other aspects are also considered, such as the reactants (precursors, stabilizers, reducers, etc.), costs, time, environmental impact during the process, waste generated, materials, and equipment.

Most materials implemented for reducing and degrading 4-nitrophenol are hybrids resulting from combining nanoparticles (NPs) with 3D materials that serve as a matrix (support body) for the other nanomaterials. Some nanomaterials are also the product of the combination of two or more elements, materials that are not exclusively NPs.^70,71^ Nanoparticles, being the most popular nanomaterials within the study involved in working with 4-NP, have suffered extensive growth, speaking exclusively of the materials used as precursors, because there are metal-based nanoparticles, metal oxides, carbonaceous, polymeric, ceramics, combinations of two or more of those as mentioned above.

### 4.1 Carbon Dots As Organic Catalysts

Among the most used materials for the study of contaminants within bodies of water that can be considered organic, ecological, environmentally friendly, or, to a certain extent, sustainable are carbon dots (CDs); this is because they can be synthesized, through chemical reagents as well as organic raw materials, such as seeds,^72^ peels,^73^ leaves,^74^ plants,^75^ etc. The use of proteins,^76^ such as enzymes,^77^ has also been increasing because they are used as a support, catalyst agent, or support material in functionalization with CDs or NPs to detect contaminants, drug delivery, etc. Table 3 presents nanomaterials of organic origin and their application and compounds that use carbonaceous structures.^78,79^

**Table 3:** Biogenic Nanomaterials for the Reduction/Degradation of 4-NP with NaBH_4_

**Catalyst**	**Green synthesis technique**	**Degradation/reduction (%)**	**Ref**
Palladium nanoparticles (PdNPs)	*Matricaria recutita*	91.4	Ahmad Malik et al.^80^
Bimetallic nanoparticles (Ag–Fe)	*Salvia officinalis*	94.6	Ahmad Malik et al.^81^
Bimetallic nanocomposites (Ag–Au)	*Hojas de té*	NR^a^	Chun-Won Kang and Haradhan Kolya^82^
AgNPs	*Tapioca starch*	95	Kalantari et al.^83^
AuNPs	*Polen de miel de abeja*	NR^a^	Kumar et al.^84^
Nanocomposite (Fe_3_O_4_@SA–CMC–CuNPs)	NR	95	Helmiyati et al.^85^
Nanocomposite (AgNPs/rGo)	*Mangifera indica*	94	Kolya Haradhan et al.^86^
AuNPs	*Solieria tenuis*	95	Wang et al.^87^

a NR= Not reported.

### 4.2 Inorganic Catalysts

Nanoparticles are mostly considered inorganic nanomaterials, and this is due to the types of reagents and synthesis methods that are implemented for their preparation. For example, metal-based nanoparticles, such as AgNPs, can be used either individually,^88^ doped with some other element or elements,^89^ in bimetallic structures,^90^ or on surfaces such as thin films;^91^ gold nanoparticles (AuNPs)^92^ and palladium (PdNPs)^93^ are other examples of metallic NPs with excellent use for this type of application. Similarly, nanomaterials such as NPs derived from metal oxides are another example.^94,95^ Some nanomaterials are classified as nanowires, nanoreactors, nanosheets, and thin and ultrathin films, to name a few, which have also been attempted to be implemented in the degradation of contaminants, dyes, and metal ions, as observed in Table 4.

**Table 4:** Inorganic Nanomaterials for the Reduction/Degradation of 4-NP

**Catalyst**	**Synthesis method**	**Degradation/reduction method**	**Degradation/reduction (%)**	**Ref**
Fe_3_O_4_@Alg-AuNPs	Solvothermal	Langmuir–Hinshelwood model	99	Ghorbani-Vaghei et al.^96^
Bi_2_O_3_	Coprecipitation	Photocatalytic (visible light)	100	Muersha and Pozan Soylu^97^
TiO_2_ nano powder	Sol–gel	Photocatalytic (visible light)	35	Muersha and Pozan Soylu^97^
ZnO nano powder	Microwave assisted coprecipitation	Photocatalytic (visible light)	34	Muersha and Pozan Soylu^97^
ZrO_2_ nano powder	Microwave assisted coprecipitation	Photocatalytic (visible light)	22	Muersha and Pozan Soylu^97^
AgNPs/Fe_3_O_4_	Coprecipitation and ultrasonic	Catalysis assisted NaBH_4_	99	Tadele Alula M, et al.^98^
Ti/TiO_2_–NiO_2_ electrodes	Humidity impregnation	Electrochemical	95	Fadillah et al.^99^
Ir/CeO_2_	Precipitation	Photoreduction assisted Na_2_SO_3_	84	Castan ~eda et al.^100^
AuCu aerogel	*In situ* reduction	Catalysis assisted NaBH_4_	96	Qin et al.^101^
AgBiO_3_ nanoparticles	Hydrothermal	Photocatalytic (metal halide lamp)	90	Boruah et al.^102^
NRGO-CoWO_4_–Fe_2_O_3_ nanocomposite	Microwave	Catalysis assisted NaBH_4_	98	Mohamed et al.^103^
PVA/AgNPs nanocomposite	Laser ablation	Catalysis assisted NaBH_4_	95	Mostafa and Menazea^104^
Pd@PPMx microspheres	NR	Catalysis assisted NaBH_4_	96	Sohail-Bashir et al.^105^
BiVO_4_/CuO hybrid material	Thermal decomposition	Photoelectron degradation	50	Martiniamo do Prado et al.^106^
ZrO_2_/g-C_3_N_4_ nanocomposite	Ultrasonic	Photodegradation (simulated solar light)	NR^a^	Zarei et al.^107^

a NR= Not reported.

As can be seen in Tables 3 and 4, the use of different raw materials, as well as the use of various synthesis methods and processes used for the reduction or degradation of 4-NP, are coarse, each differing from the others due to very minimal issues such as the time used, the concentration of NaBH_4_, volume of water, amount of catalyst used. Some parameters differentiate them in a more complex or complicated way, such as the process by which the reduction is carried out.

### 4.3 4-Nitrophenol Reduction Methods

The 4-nitrophenol reduction reaction is a well-known probe followed by 4-aminophenol (4-AP) formation. 4-AP is the final intermediate in the industrial production of acetaminophenol (paracetamol). As per our recent finding, we can also produce hydroquinone and phenol by changing the pH of the solution after the 4-aminophenol formation. These are also used as a standard depigmentation, disinfect skin and freckles to relieve itching, and postinflammatory hyperpigmentation as an anesthetic in products.

**Figure 5:**
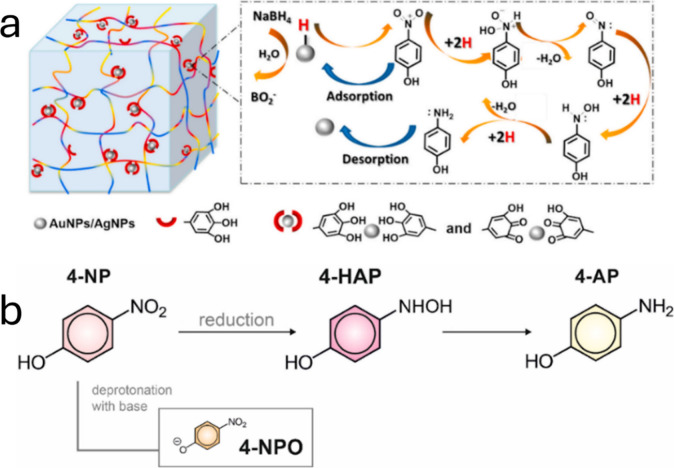
Catalytic reduction reaction mechanisms: (a, b) 4-NP reduction with NaBH_4_ and using different catalysts. Adopted with permission from refs (131 and 132). Copyright 2021 Elsevier and 2020 MDPI.

The processes by which the reduction or degradation of different pollutants found in air, soil, or water bodies are carried out are usually through catalysis or electrochemical processes, and this is because these types of processes show a high degree of efficiency, effectiveness, low costs, and mild reaction conditions, that is, they do not generate a reaction that could become dangerous before, during, or after the process. Catalysis is a chemical process consisting of accelerating or modifying chemical reactions through a substance called a catalyst. The types of catalysis are differentiated by the phase in which the catalyst and the different reactants are occupied (homogeneous (catalysis includes all those systems in which the reactants, products, and catalyst are in the same phase), heterogeneous (unlike homogeneous, in heterogeneous catalysis the reactants and products are in a different phase than the catalyst), enzymatic (catalysis in which the catalyst used is biological, mostly protein catalysts, it can be a catalysis homogeneous or heterogeneous) as shown in Figure 5) or from the source where the energy with which the chemical reaction is carried out comes (photocatalysis, electrocatalysis, and thermocatalysis, to mention a few). Adsorption, surface reaction, and desorption are the reaction mechanisms, which will differ between reactions where different catalysts are used, a different medium is present, or it is carried out with a different energy source.^116^

#### 4.3.1 NaBH_4_-Based Reduction

Catalysis is a fundamental concept in chemistry that plays a crucial role in facilitating and accelerating chemical reactions. It is defined as the process by which a substance called a catalyst modifies the rate of a chemical reaction without undergoing any permanent change. Catalysis provides an alternative pathway for a reaction to occur more quickly than under normal conditions. A catalyst is a substance that starts or speeds up a chemical reaction by reducing the activation energy required for the reaction to proceed. This means that the catalyst allows the reactants to reach the transition state more quickly, making the reaction more favorable. In catalysis, intermediates are temporary species that form during a reaction but are absent in the final products. These intermediates are crucial for understanding catalytic processes’ reaction mechanisms and steps.^117^

The principles of catalysis encompass several aspects of this essential chemical phenomenon:

1. Catalyst functionality: Catalysts provide an alternative reaction pathway with lower activation energy. After the reaction, they remain unchanged in their chemical composition and can be reused.
2. Measurement of catalytic activity: Catalytic activity is quantified using the turnover frequency (TOF), which represents the number of reactions a catalyst can facilitate per unit of time.
3. Pathways and mechanisms: Depending on the catalyst and reaction, catalysis can involve various mechanisms, such as acid–base catalysis, enzymatic catalysis, and heterogeneous catalysis.
4. Homogeneous/heterogeneous catalysis occurs when the catalyst and reactants are in the same phase (e.g., liquid or gas). In contrast, heterogeneous catalysis involves a catalyst in a different phase than the reactants. (e.g., solid catalyst in the gas phase).

Different research groups have incorporated the catalysis process for their studies on reducing 4-NP. For example, Ghorbani-Vaghei and his collaborators used a compound of Fe_3_O_4_ nanoparticles attached to sodium alginate through sonication of the nanoparticles in a solution at a concentration (0.5% w/w) for 30 min, the catalytic process occurred with the use of 1.0 mg of Fe_3_O_4_@Alg-Au NPs dispersed in 3.0 mL of 2.5 mM aqueous solution of 4-NP with a small diluted solution of NaBH_4_ (2.5 × 10^–4^ M), this solution was at room temperature, this reaction was followed by UV–vis spectroscopy where it initially presented a yellow color and at the end presented a white/transparent color (characteristic color of 4-AP), they carried out characterization of the nanomaterial using FT-IR, XRD, TEM, SEM, EDX, and VSM, finally obtaining a reduction of 99%.^96^ Likewise, Alula and company synthesized Fe_3_O_4_ nanoparticles and AgNPs using the ultrasonication method, creating a compound of these for use in the reduction of 4-NP.^98^ The compound was characterized using TEM, XRD, Seira, and UV–vis spectroscopy. The reduction mechanism they implemented was based on the preparation of a water solution, NABH_4_, and nanomaterial solution (AgNPs/Fe_3_O_4_), which was measured during its UV–vis spectroscopy process, where a 99% reduction of 4-NP present in the solution is shown.

##### Reaction Mechanisms

Different reaction mechanisms have been reported as a result of using various materials for the reduction or degradation of 4-NP, one of the most common and used for more than 100 years and currently is the reduction of 4-Nitrophenol (4-NP) to 4-Aminophenol (4-AP) through an aqueous solution in which sodium borohydride (NaBH_4_) is used, which is a reducing agent^120^ (Figure 5). Guangyu Wu and his work team 2018–2019 developed a mechanism for the reduction of 4-NP to 4-AP, using hybrid vesicles doped with AuNPs gold nanoparticles in an environment prepared by the combination of 4-NP, NaBH_4_, deionized water, and hybrid structure, giving an efficiency of almost 100%, as reported.^121^ Most of the works reported in recent years dedicated to the degradation or reduction of 4-NP use reaction mechanisms where sodium borohydride NaBH_4_ is one of the main protagonists, as shown in Figures 5. The factors that change the catalytic efficiency,^122,123^ the nanomaterial,^124,125^ the reaction time, and the percentage of effectiveness.

#### 4.3.2 Electrocatalysis

Electrocatalysis is a catalytic process that involves oxidation/reduction through electron transfer. This branch involves the field of chemistry along with other related disciplines. In electrochemical systems, Electrocatalysis refers to all those reactions that occur at the interface between the catalyst and an electrolyte solution that serves as the medium where the reaction will occur. The main objective of this type of process is to increase the reaction rate, obtain the desired products, and ensure that the catalyst does not change. This type of mechanism is essential in industrial fields and bodies of work dedicated to research to improve in areas such as environmental protection and remediation, energy systems, and chemical product systems.

**Figure 6:**
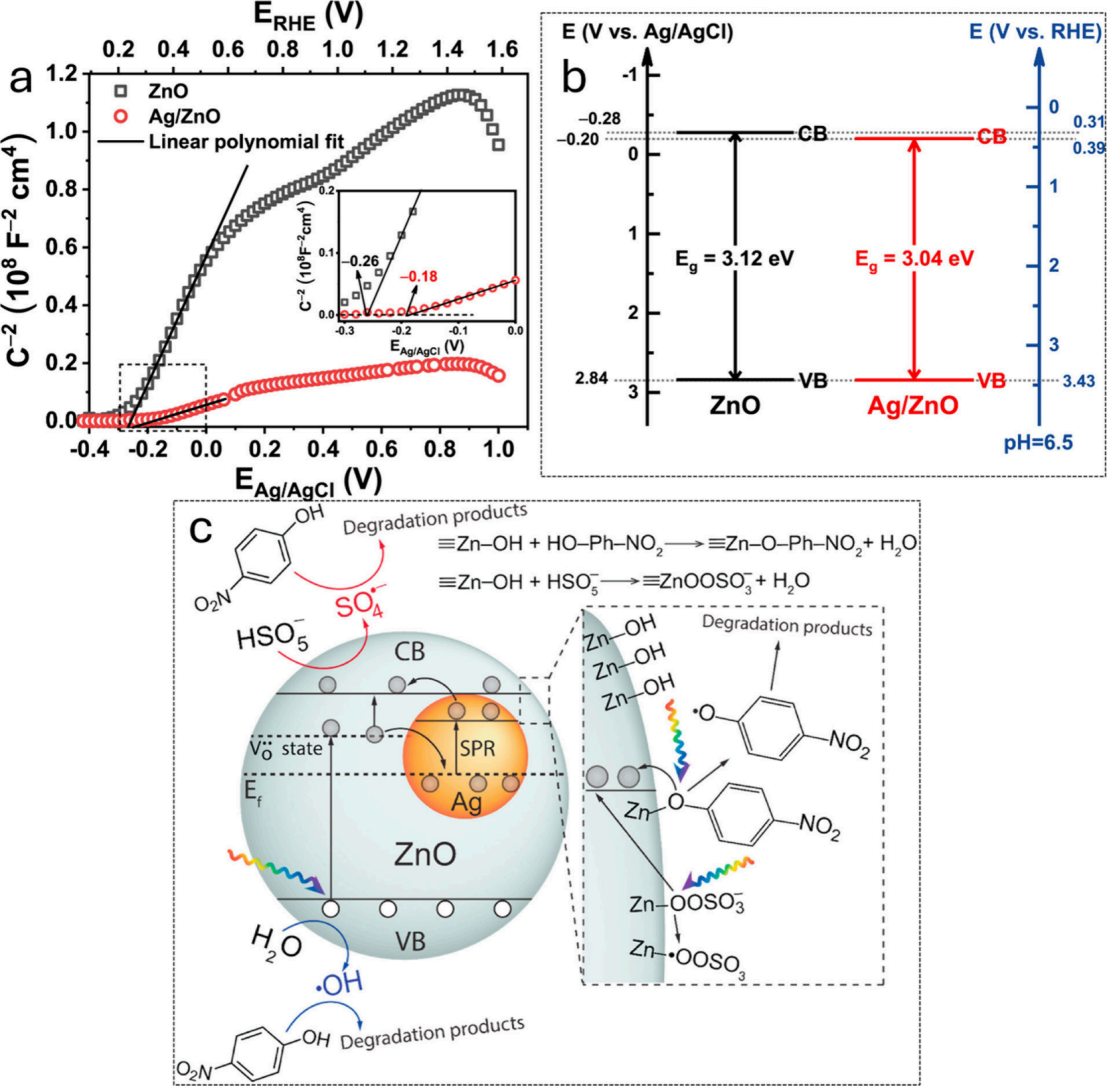
(a) Mott–Schottky plots collected in dark at 1 kHz, (b) band gap illustration, and (c) possible mechanism for the 4-NP degradation in the photocatalyst (Ag/ZnO/PMS/Vis) system [^123^]. Copyright 2021 Elsevier.

Ganjar Fadillah and his working group (2019) synthesized Ti/TiO_2_–NiO electrodes to conduct experiments on electrochemical processes focused on reducing 4-NP. The experiment used two electrodes system. The conversion of 4-nitrophenol was carried in NaCl and H_2_O_2_ electrolyte solutions. The pH-dependent and reaction times conditions were optimized to construct ideal conversion of 4-NP molecules. The cyclic voltammetry method measured the degradation of 4-NP solution in the potential range of 0 to −1 V with a scan rate of 0.050 V/s. This experimentation resulted in a 95% effectiveness in reducing the 4-NP^99^ (Figure 6).

The electrocatalytic 4-NP reduction activity was confirmed through cyclic voltammetry (CV) followed by electrochemical surface area (ECSA), Tafel slope, and electrochemical impedance spectroscopy (EIS) measurements. The proposed mechanisms are based on the redox peak (≤−0.8 V vs Ag/AgCl) in the CV curve of the catalyst, and the coordination of water molecules around the catalyst and nitro group (NO_2_) of 4-NP through this catalyst can induce the formation of H-bonds between them. It is also confirmed that the distorted stretch vibrations of the NO_2_ drastically altered 6 cm^–1^ when the 4-nitrophenol molecule onto the electrocatalyst. Furthermore, the authors reported that the electrocatalytic activity rests on the number of H-bonds presented in the reaction system, and the electrons from the catalyst could efficiently be introduced into the 4-NP, followed by the production of 4-AP. Further, P. S. Ong et al. performed mass spectroscopy analysis after the 4-nitrophenol reduction, interestingly the authors found that apart from the formation of 4- AP (110.06 Da), p-benzoquinone imine (108.04 Da), 4,40 -Dihydroxyazobenzene (215.08 Da), semiquinonimine radical derivative (109.05 Da), and two dimers, 4-Amino-3-[(4-hydroxyphenyl) amino] phenol (peak at *m*/*z* of 217.2 Da) and (3-[(4-Hydroxyphenyl)amino]-4-imino-2,5-cyclohexadien-1-one) (215.12 Da) side reaction reactions A and B were found.

#### 4.3.3 Photocatalysis

The photocatalysis was assessed by decomposing 4-nitrophenol (4-NP) under visible light exposure in ambient conditions. Each photocatalytic test involved dispersing a certain amount of the catalyst in a liquid phase mixture of 4-NP. Generally, the photocatalytic experiments were conducted in a flask designed to optimize lighting performance and equipped with a heat dissipation procedure to avoid hotness. A spotlight photoreactor was utilized, emitting light from 303 to 518 nm with 18 cm from the lamp to the sample mixture. Before light exposure, the solution was stirred in darkness for 60 min to achieve adsorption stability among the photocatalyst and 4-NP. In 45 min of treatment, samples were periodically withdrawn and filtered through a PTFE filter (450 nm pore size). The concentrations of 4-NP and its degradation intermediates were analyzed using liquid chromatography. To address catalyst reusability, recovered catalysts were separated via centrifugation and reused with fresh 4-NP solutions. Experimental conditions remained constant across five fresh 4-NP solutions, ensuring consistent catalyst performance evaluation.^97^

In technical terms, photocatalysis is the combination of photochemistry with catalysis. This mechanism generates e^–^–h^+^ pairs and free radicals, for instance hydroxyls (·OH),^108^ which participate in secondary reactions. The main characteristic of photocatalysis is that solar energy is converted into chemical energy, which similarly interacts with the catalyst and the electrolyte solution. Wusiman Muersha, together with Gulin Selda Pozan Soylu, synthesized catalysts were tested for the degradation of 4-NP using semiconductors, giving rise to nanopowders first of Bi_2_O_3_ which was synthesized by the coprecipitation method using Bi(NO_3_)_3_ 5H_2_O, NaOH, nitric acid as reagents, in the same way, they synthesized zinc oxide (ZnO) and zirconium oxide (ZrO_2_) powders by the microwave-assisted coprecipitation method and titanium oxide precipitates by the sol–gel method.^97^

The possible photocatalytic 4-nitrophenol reduction mechanism is based on the nitro group (NO_2_), which can alleviate the 4-phenolate ion (4-NP^–^) by moving the O^–^ → H_2_ to develop the amine group (NH_2_). The proposed mechanism for this photocatalytic conversion is based on the following steps (Figure 6): (1) initially, 4-nitrophenol can convert into 4-nitrophenolate ion in the presence of a basic pH medium followed by the adsorption onto the photocatalyst owing to the electronegativity of the NO_2_. (2) Further, the photocatalyst must satisfy Jablonski’s theory, i.e., the photon’s energy is higher than the bandgap of the catalyst material. In practice, the photocatalyst is exposed to the lamp to make unpaired electrons in the ground state (VB) excited by a photon into a higher energy vibrational state (CB), and the photocatalyst loses some energy with the emission of phonons, lattice vibrations, and thermalization. (3) This electron relaxes back to the VB, releasing a lower energy photon (than excited energy) through recombining an electron and hole pair. The metallic semiconductor-based photocatalysts’ particles can trap photogenerated electrons through the Schottky barrier generated between the metal–semiconductor interface. Trapped photogenerated electrons are transferred to the adsorbed 4-NP on the surface of the photocatalyst, followed by the conversion of 4-NP to 4-AP as the final product.

#### 4.3.4 Thermocatalysis

Thermocatalysis is a process in which temperature performs a critical function in the speed of a chemical reaction. In this case, the temperature’s heat activates the catalyst, accelerating the reaction rate. Unlike common catalysis, which does not depend directly on temperature, thermocatalysis is based on thermal energy. This process is widely used in hydrogenation reactions (ammonia production, Fischer–Tropsch synthesis), thermal decomposition, and reforming reactions (oil industry).

Xiaoqing Liu and his collaborators and work team synthesized a Na_2_Ta_2_O_6_ compound doped with silver nanoparticles (AgNPs). The hydrothermal method is used to continue with a chemical reduction process. This composite material was subjected to thermocatalytic tests to reduce nitrobenzenes (NB) to amino benzenes (AB). In practice, 4-NP was used as nitrobenzene, and its reduction to 4-AP as aminobenzene with borohydride in the aqueous solution, the working temperature range was from 25 to 65 °C passing through 35°, 45°, and 55°, giving a degradation of the contaminant of 99%.^119^ Additionally, parallel experiments were conducted under similar conditions but with increased quantities to validate the catalytic reaction’s accuracy.^119^

The projected understanding for the reduction of 4-NP thermocatalytic way is similar to traditional catalysis: (1) the catalyst active spots and the charge conglomeration at the catalyst surface significantly contributed to the 4-NP reduction reaction through the electron donor addition of a potent reducing agent (NaBH_4_), with the addition of borohydride solution the pH of the medium altered to basic and the 4-NP is converted to 4-nitrophenolate ion through deprotonation process. (2) The surface interface with NO_2_ with H^+^ is present on the metal catalyst surface, followed by the alteration of 4-nitophenolate ion to 4-aminophenol through different intermediate product formations through the hydro-deoxygenation reactions. (3) The higher temperature in the reaction mixture leads to colloid molecules at a higher rate with more force, which leads to a faster reaction rate.

As has been observed, various methods and materials are used to reduce 4-NP. Despite the variety of existing methods and nanomaterials, the various scientific investigations analyzed share similar or identical aspects. For instance, NaBH_4_ is the predominant catalysis process over others (electrocatalysis, thermocatalysis, and photocatalysis), and the notable presence of metals or metal oxide nanomaterials as the catalyst material for most of the studies employed. Proposing a method and a single nanomaterial or some hybrid that can be confidently affirmed as the best option above all others is a very complex task today. Critical criteria must be satisfied: low cost, high efficiency, effectiveness, reusability, minimal environmental impact (or none), rapid synthesis, accessibility, and more.

Nevertheless, the sunlight-driven photocatalytic process is recommended due to the minimal chemicals used. However, there is still a need to improve the understanding of the mechanisms and byproduct analysis. Similarly, the photocatalysts must convince the industrial scale usage through sustainability, simple procedures, higher catalytic removal efficiency, and reproducibility.

### 4.4 Theoretical Perspective

**Figure 7:**
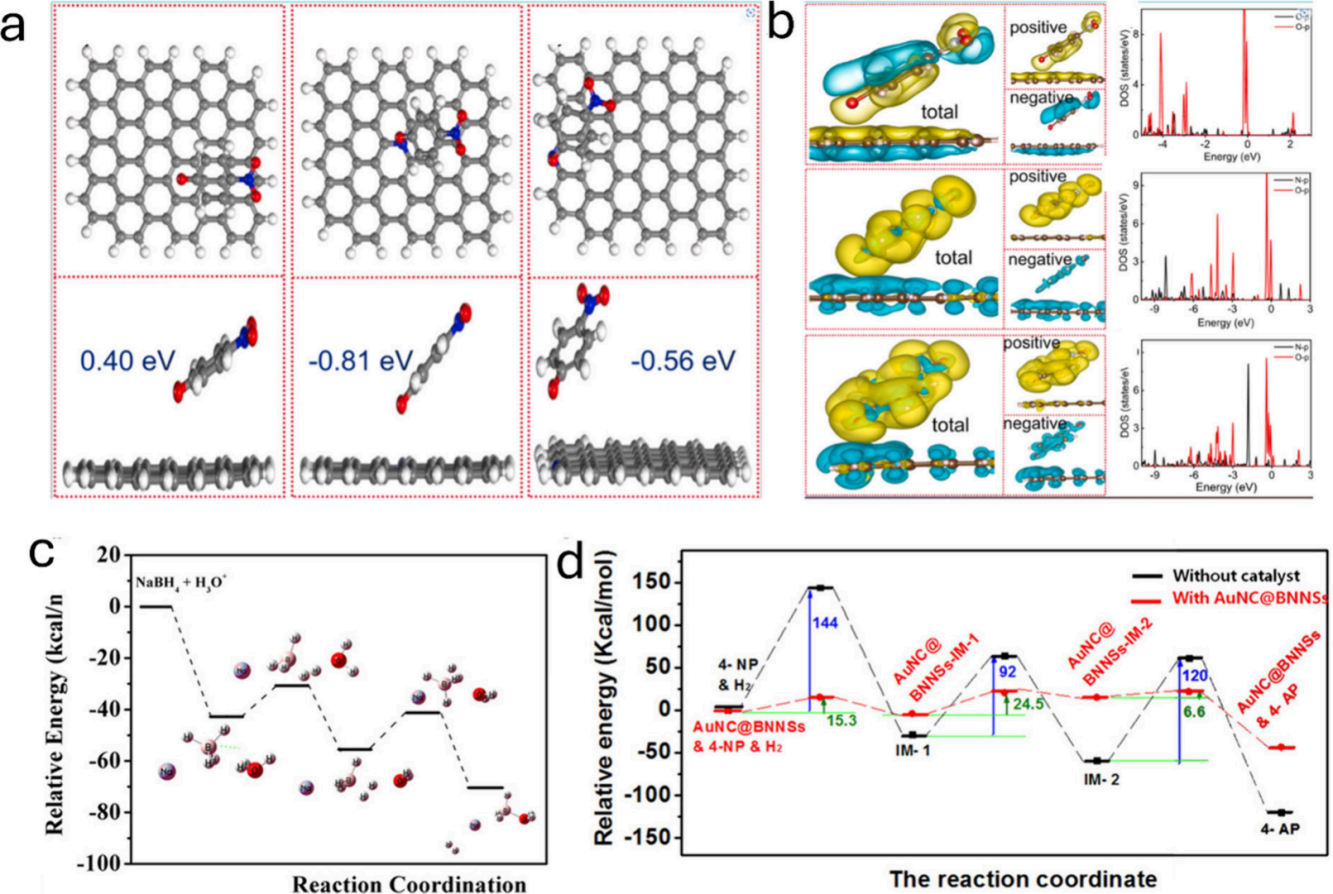
(a, b) Optimized structures and charge density and PDOS of the soley graphene (left), graphitic-N graphene (center), and pyridinic-N graphene (right) with 4-nitrophenol.^135^ (c, d) Catalytic response route and energy sketch of the catalytic process with and without catalyst^136^ Adopted with permission from refs (135 and 136). Copyright 2021 Elsevier and 2019 Asian Chemical Editorial Society.

There are few theoretical works to understand the reduction mechanism, but there have been significant advances (Figure 7). Abu-Dief et al.^133^ synthesized Ag-NPs using M. oleifera and Delonix regia as precursors, effectively reducing the 2,4-DNF to 2,4-DAF. In addition, they could predict the reaction mechanism through DFT calculations. Through theoretical calculations, Thanthrige et al.^134^ observed that the 4-NP molecule binds to silver favorable to the −NO_2_, creating a program to convert −NO_2_ into −NH_2_. Chen et al.,^135^ who synthesized nitrogen-doped porous carbon through algae and DFT calculations, revealed the key character of the N atoms into the carbon structure to catalyze this reaction by adjusting the carbon atoms electronic configuration followed by endorsing the adsorption of 4-nitrophenolate ions on the doped carbon material. Liu et al. demonstrated this through DFT calculations with molecular hydrogen rather than borohydride.^136^ Furthermore, they supported their improved results because of the continual e^–^ move from the 4-nitrophenolate ion to boron nitride nanosheets decorated with gold particles (Figure 7).

### 4.5 Parameters that Influence Detection and Degradation/Reduction

#### 4.5.1 4-Nitrophenol Detection

1. *Dynamic range*: The detection method’s range of concentrations can provide accurate measurements. A wide dynamic range allows the recognition of contaminants.^137^
2. *Sample matrix*: The sample matrix refers to the components present in the sample and the contaminant of interest. The matrix’s composition can affect the detection method’s effectiveness and may require sample pretreatment to eliminate interferences.^138^
3. *Chemical compatibility*: Chemical compatibility between the sample and the reagents used in the detection method is essential to avoid unwanted reactions that may affect the results.^139^

In addition, two more factors are found, which are crucial when detecting 4-NP: temperature and pH. These are two important factors that can influence the detection of contaminants at the laboratory level in several ways:

1. *Effect on the stability of contaminants*: Temperature and pH can affect the stability of contaminants in the sample. Some contaminants can decompose or chemically react with other sample components under specific temperature or pH conditions, making them difficult to detect or alter measurement results.
2. *Interference with detection methods*: Temperature and pH can interfere with detection methods used in the laboratory. For example, changes in pH can affect the net charge of molecules and, therefore, influence their affinity for specific reagents or probes used in detection assays. Similarly, temperature can affect the rate of chemical reactions used in some detection methods, influencing the sensitivity and accuracy of measurements.
3. *Instability of reagents and equipment*: Extreme temperature or pH conditions can affect the stability of the equipment used in the laboratory. For example, some reagents may degrade more rapidly at elevated temperatures or in acidic or alkaline media, which can affect the accuracy and reliability of measurements.
4. *Interference with the sample matrix*: Temperature and pH can also influence the composition and properties of the sample matrix. Changes in temperature or pH can alter the solubility of sample components and their ability to interact with contaminants of interest, which can influence the effectiveness of extraction, purification, and detection methods used in the laboratory.^140^

#### 4.5.2 4-Nitrophenol Reduction

1. *Type of catalyst*: As we already saw, many nanomaterials can be used as catalysts, each with their own advantages and disadvantages.^137^
2. *Size and shape of nanomaterials*: The dimensions of nanomaterials can influence their catalytic activity. Specific sizes can provide greater surface area and a more remarkable ability to interact with 4-nitrophenol molecules.^141^
3. *Catalyst concentration*: The amount of nanomaterial used must be controlled during the catalysis process because the reaction’s speed depends on the catalyst’s amount. Without concentration control, unwanted side effects or reactions may arise.^138^
4. *Temperature*: Temperature is also a source of energy that can affect the catalysis process (if not used with control), allowing secondary reactions to arise, and damaging the catalyst, reactants, and therefore, the products.^138^
5. *Active surface area*: It is crucial, the larger the surface area, the more active reactive sites are available for the reaction. Nanomaterials with a high surface-to-volume ratio are more effective.^138^
6. *pH conditions*: The medium’s pH influences the nanoparticles’ surface charge and their ability to interact with 4-nitrophenol.^138^
7. *Effect of light*: Some nanomaterials can be photosensitive, and exposure to light can influence their catalytic activity.^138^
8. *Crystalline structure*: The crystalline structure of nanomaterials also plays a role. Different crystal faces can have different catalytic properties.^138^
9. *Nanomaterial stability*: The stability of the nanomaterial over time is important to maintain its catalytic activity.^138^
10. *Chemical stability*: The chemical stability of nanomaterials in the reaction medium is crucial. Some nanomaterials can degrade over time.^139^

## 5 Conclusion and Future Perspectives

The status of identifying and removing the contaminant 4-nitrophenol has experienced significant advances thanks to continuous research and development of technologies. Regarding identification, investigative procedures have been improved to recognize and quantify 4-nitrophenol in the environment, including methods such as UV–visible spectroscopy, electrochemical, and even surface plasmon resonance with optical fiber, with which picomolar quantities could be identified. In terms of removal, several effective methods have been developed. Bioremediation, which uses microorganisms to degrade 4-nitrophenol, has proven to be a promising option. Additionally, chemical oxidation with agents such as hydrogen peroxide or ozone and adsorption on materials such as activated carbon are also used to remove contaminants from water and other media.

As for the future perspective, research is expected to continue to focus on improving the efficiency and cost-effectiveness of 4-nitrophenol identification and removal methods. This could involve the progress of more advanced and eco-friendly methods, as well as the optimization of existing processes, so more theoretical research is required to understand at a molecular level the process of identification and removal of 4-NP, as well as an approach more accurate, in which the effect of natural waters (taps, rivers, lakes, and sea) on the different methods and materials proposed for the detection and removal of 4-NP is investigated. Additionally, it is expected that there will be greater integration of preventive approaches to address the release of the contaminant into the environment. This could include implementing cleaner industrial practices and developing safer, more sustainable alternatives to processes that use or produce 4-AP. Despite significant progress, the 4-NP identification and removal field remains dynamic and developing. Research and innovation are expected to continue improving our capabilities to address this pollutant and protect human health and the environment.

## Data Availability

The data that support the findings of this study are available from the corresponding author upon reasonable request.
